# Skull Metastasis Extending to the Superior Sagittal Sinus: An Unfamiliar Presentation of Papillary Thyroid Carcinoma

**DOI:** 10.7759/cureus.2738

**Published:** 2018-06-05

**Authors:** Abu Baker Sheikh, Aisha Akhtar, Usman Tariq, Abdul Ahad E Sheikh, Fasih Sami Siddiqui, Marvi M Bukhari

**Affiliations:** 1 Internal Medicine, University of New Mexico, Albuquerque, USA; 2 Department of Surgery, Texas Tech University Health Sciences Center, Lubbock, USA; 3 Research Assistant, Yale University School of Medicine, New Haven, USA; 4 Student, Shifa College Of Medicine, Islamabad, PAK; 5 Internal Medicine, Shifa College Of Medicine, Islamabad, PAK

**Keywords:** papillary thyroid carcinoma, metastasis, humerus fracture, superior sagittal sinus invasion

## Abstract

Thyroid cancer is the most common endocrine cancer in the world, with a rising global incidence over the last three decades. Papillary thyroid cancer (PTC) is the most common type of thyroid neoplasia, accounting for 74%-80% of all cases. Skull metastasis from a differentiated thyroid malignancy is a rare occurrence, while a subsequent dural involvement is even more inimitable. As such, a clinician requires a high degree of clinical suspicion and resultant radiographic evidence in order to make the diagnosis. Here we present the case of a 54-year-old male patient who presented with a pathological fracture of his right humerus, a midline frontal bone swelling and an asymptomatic neck mass. Further workup revealed follicular variant papillary thyroid carcinoma (FV-PTC) with distant metastasis to the calvarium. The conventional therapy for metastatic PTC includes a total thyroidectomy, removal of resectable metastatic lesions and a supplementation with radioactive iodine (RAI) and/or external beam radiation at the sites of the metastases. This case and our literature review illustrate that skull metastases should be considered in the clinical course of PTC so that appropriate management can be started.

## Introduction and background

Thyroid cancer is the most prevalent endocrinal malignancy in the world, with an incidence that continues to rise worldwide in the last three decades. Papillary thyroid cancer (PTC) is the most common variant of this malignancy and accounts for 74%-80% of all its presentations. PTC usually has a slow progression and is associated with a regional spread to the cervical lymph nodes. It carries a favorable outcome, except in the presence of a metastatic disease process [1]. Distant metastases (DM) occur in approximately 10% of the patients with a PTC, with the lung and bone being the most commonly perpetrated sites [2, 3]. While the follicular variant of a papillary carcinoma has a greater propensity for bony metastasis, distant metastasis from a primary PTC to the calvarium and a subsequent encroachment of the brain is a very rare clinical predicament. We present a case akin to this metastatic distribution that presented as a large soft tissue mass on the head.

## Review

Case presentation

A 54-year-old male with multiple comorbidities including hypertension, hyperlipidemia, type II diabetes mellitus and stage II chronic kidney disease, presented to our facility with complaints of a gradually enlarging frontal bone mass for the last one month, as well as progressive right arm pain which exacerbated with movement, following a malunion fracture of his right humerus. He also had a painless neck swelling for the last two years which went unreported, owing to a lack of any associated symptoms such as dyspnea, dysphagia or hoarseness.

Initial assessment suggested that he was in good physical health with a heart rate (HR) of 82/minute, respiratory rate (RR) of 18/minute with 97% oxygen saturation, a temperature of 98.4°F and blood pressure (BP) of 112/75 mm Hg. He was found to be well oriented with intact responses to an extensive cranial nerve exam. The swelling in the left frontal region of the head was easily discernable. It measured 6 cm x 7 cm, soft and warm to the touch with a normal overlying skin but a palpable erosion of the underlying bone. A concomitant examination of his neck divulged a large left thyroid mass which measured 8 cm x 6 cm. It was noted to be hard and fixed to the underlying soft tissue structures and led to a resultant right sided deviation of his trachea. His cervical lymph nodes were not palpable across all subgroups.

**Figure 1:**
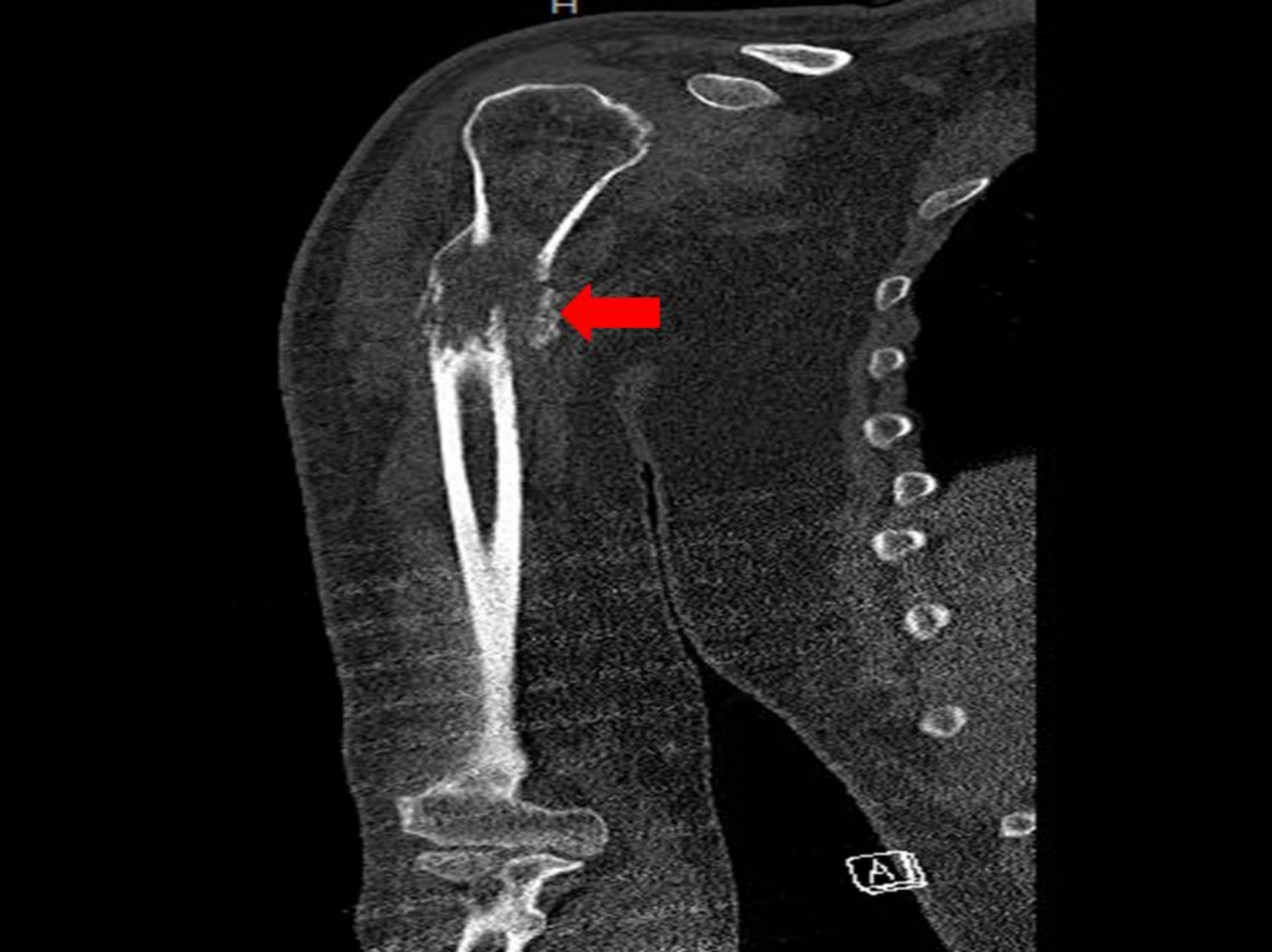
Computed tomography scan of the right arm showing a mass lesion (red arrow) in the proximal diaphysis of the humerus, invading the intramedullary canal and extending beyond the boundaries of the humerus.

He was further investigated with an X-ray of his right arm which revealed lytic lesions. A subsequent computed tomography (CT) scan of his right upper extremity (Figure 1) showed the existence of an aggressive neoplastic process in the proximal diaphysis of the humerus that had invaded the intramedullary canal, leading to the expansion of the cortex with a resultant erosion which culminated to a complete pathological fracture.

**Figure 2:**
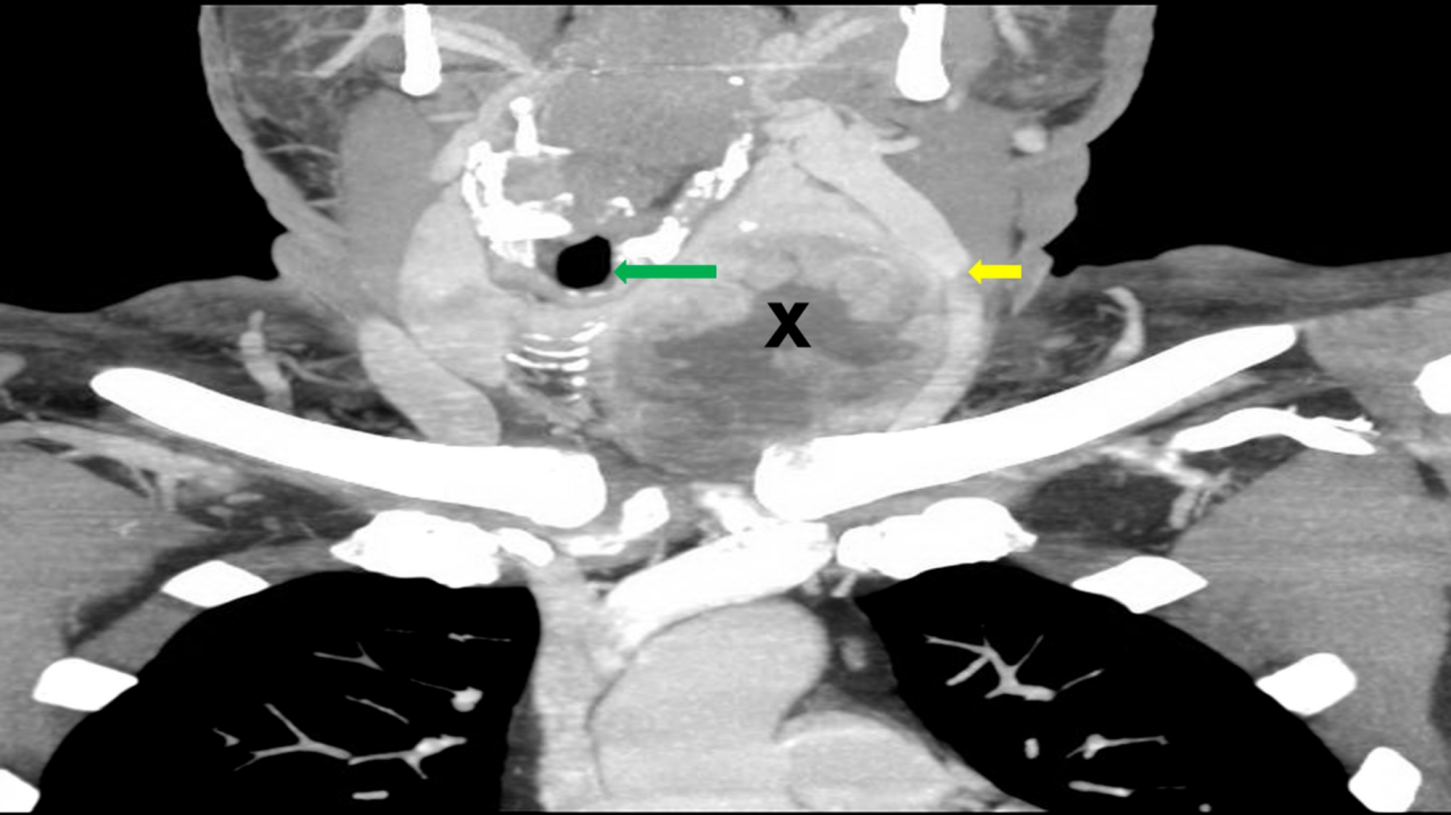
Computed tomography scan of the neck showing a necrotic thyroid mass (labeled X) on the left side, extending into the anterior mediastinum, and compressing the surrounding structures including the internal jugular vein (yellow arrow). Also visible is the trachea (green arrow) which is deviated towards the right side.

A CT scan of the neck (Figure 2) showed a necrotic mass in the left thyroid gland that measured 6.7 cm x 8.4 cm and extended into the anterior mediastinum leading to the compression of vital vascular structures such as the left carotid artery and the left internal jugular vein.

**Figure 3:**
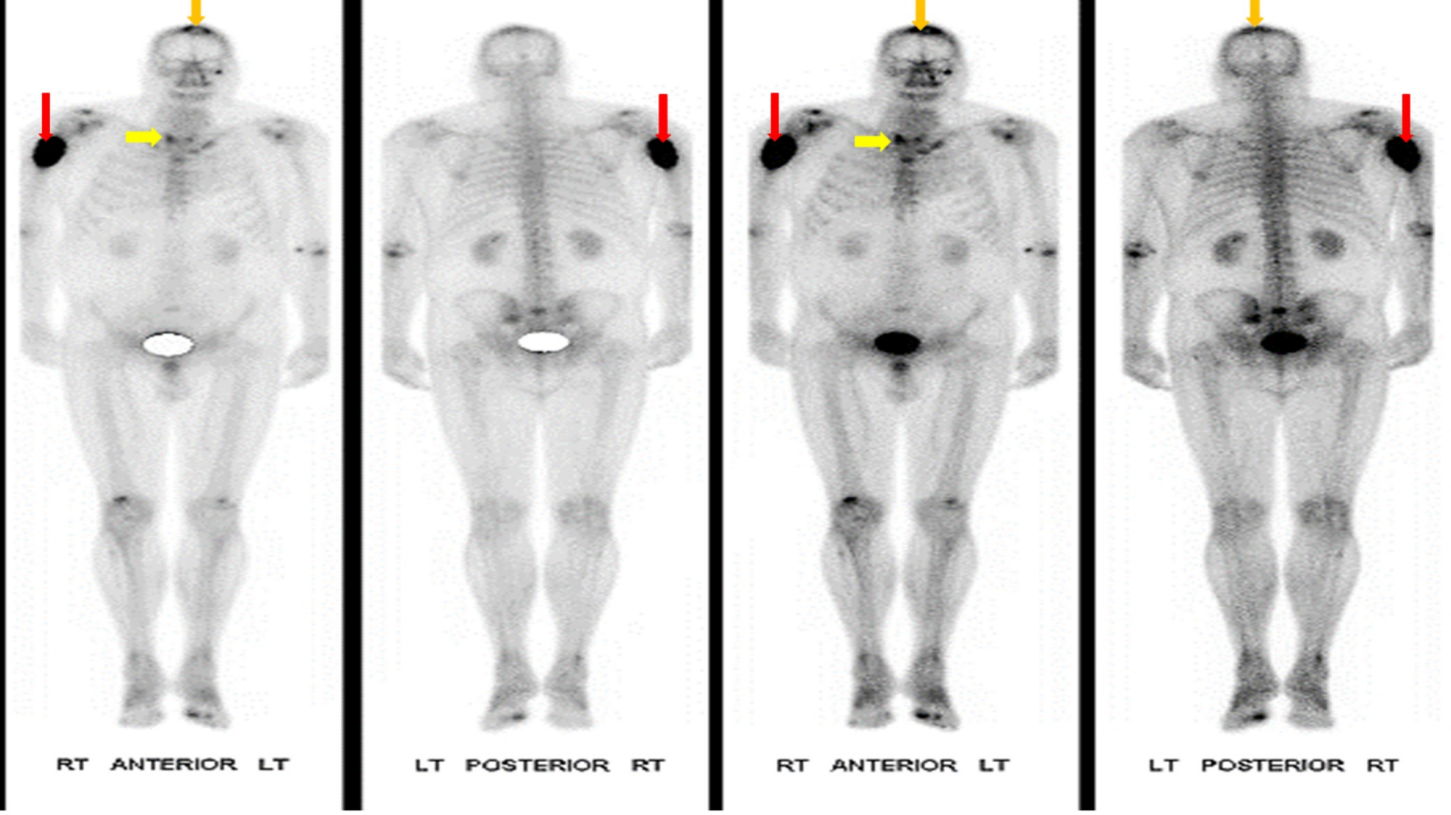
Bone scintigraphy of the patient, showing an increased uptake in the right proximal humerus (red arrows) as well as the apex of the skull (orange arrows). There is also an increased uptake in thyroid gland (Yellow arrows).

The tumor had well-defined margins but was not associated with cervical lymphadenopathy. In order to establish a tissue diagnosis, the patient was subjected to a fine needle biopsy (FNA) and a CT guided biopsy of the mass, which revealed a metastatic papillary carcinoma of the thyroid gland. Immunohistochemistry further revealed it to be positive for Thyroid Transcription Factor 1 (TTF-1), Vimentin and Cytokeratin 7 (CK7) and negative for Napsin A, carcinoembryonic antigen (CEA), prostate-specific acid phosphatase (PSAP), prostate-specific antigen (PSA), Cytokeratin 20 (CK20) and CDX2 tumor markers. The patient was further analyzed for a metastatic disease via a bone scintigraphy (Figure 3) which showed an increased uptake in the proximal humerus and the apex of the skull with an extradural effect. During the bone scan workup, there was an evident increase in the size of the swelling in his frontal bone.

**Figure 4:**
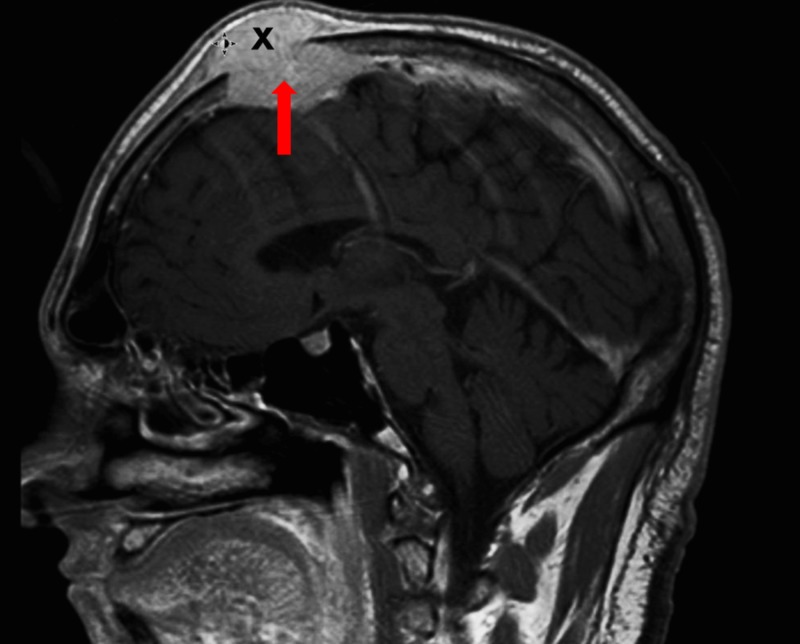
Magnetic resonance imaging of the head showing an enhancing calvarial mass (marked X) invading into the subcutaneous tissue (red arrow). Also visible is the involvement of the inner table of the calvarium, and invasion of the dura mater by the mass at the vertex.

This mass was further investigated with a magnetic resonance imaging (MRI) scan of the head (Figure 4) which revealed an enhancing calvarial mass with an extension into the subcutaneous tissue. There was also an incursion into the inner table of the calvarium with a possible invasion of the dura at the vertex. This was accompanied by a 6 cm x 4 cm x 4 cm mass that presented as a probable invasive meningioma or a metastatic lesion. The invasiveness of this mass was further assessed via a conventional cerebral angiogram which confirmed the suspicion that the mass had raided the superior sagittal sinus.

As illustrated, the patient had a myriad of clinical issues which warranted prompt surgical intervention. He first underwent a total thyroidectomy. The histological evaluation suggested that the tumor was a follicular variant of a papillary carcinoma, measuring 10 cm x 6.5 cm x 6 cm with a lack of lymphovascular invasion. Three days following the thyroidectomy, the patient underwent a bi-frontal craniectomy which included the resection of the superior sagittal sinus and part of falx cerebri along with the tumor, which measured 5.8 cm x 6 cm x 4 cm. The histological evaluation revealed a metastatic papillary thyroid carcinoma involving the dura with extensive lymphovascular invasion. The tumor cells were morphologically similar to a follicular variant of a papillary thyroid carcinoma which is a very rare clinical find. The tumor cells were negative for CK20 and CDX2 and positive for CK7, TTF1, and PAX8 markers. These findings supported the morphological impression and the above diagnosis. Cranioplasty was performed one week after the craniectomy.

His initial chief clinical complaint of a right humeral lesion was eventually addressed. The mass was excised and a reconstruction was performed with a humeral bone allograft and stabilized with a periarticular humerus plate. Following a recovery from this array of surgical interventions, the patient was started on radiotherapy. The patient is scheduled for an upcoming magnetic resonance imaging (MRI) scan and an I-131 whole body scan.

Discussion

Thyroid cancer is the most common endocrinal cancer in the world, with a rising global incidence over the last three decades [4]. Fortunately, the five-year survival rate continues to improve over the same time period and is currently perched at a strong 97.8%. This can be attributed to the fact that 70% of the cases are now diagnosed at the initial stage [5].

A frequent presentation of thyroid cancer is a neck mass which could be asymptomatic or present with troublesome features. The presence of symptoms such as dyspnea, dysphagia or hoarseness of the voice usually indicates that the tumor is aggressive and is involving the recurrent laryngeal nerve. Other symptoms such as a cough, dyspnea, and stridor suggest a tracheal impediment, secondary to the mass effect of a locally invasive tumor. Such findings accentuate the need for a thorough patient history and a comprehensive physical examination [6].

Approximately 90% of the thyroid neoplasms are differentiated thyroid cancers (DTC). This term incorporates both papillary and follicular variants [7]. Only a small proportion (2%-13%) of the patients afflicted with DTC develop bony metastasis; unfortunately, this decreases the survival rate by more than 60%. The axial skeleton is the primary site of metastatic spread, with the most common sites of metastases being the spine (34.6%), the pelvis (25.5%), sternum and ribs (18.3%), extremities (10.2%), shoulder girdle (5.4%), and the craniomaxillofacial bones (5.4%) [8]. The metastatic incursion of the skull develops in 2.5%-5.8% of the cases and mostly affects the base and the tissues that dwell within, such as the sella turcica, pituitary gland and vascular structures such as the cavernous and sphenoid sinuses [9].

The histological description of a DTC plays a pivotal role in determining patient outcomes in terms of metastatic spread and their survival. Various studies have shown that histology is the single most important predictor of mortality in patients with distant metastasis, in comparison to other variables such as age, the degree of tumor infiltration, the extent of lymphovascular invasion and the size of the tumor [10]. PTC is the most common histological variant of a DTC and accounts for 74%-80% of all its cases [3]. PTCs are mostly associated with a slow progression and regional spread to the cervical lymph nodes. They tend to carry a favorable outcome, while patients having a metastatic disease process are the usual exception [1]. A distant metastatic distribution in the setting of a PTC occurs in approximately 10% of the patients, with the lung and bone being the most commonly affected sites [2]. While the occurrence itself is rare, bony metastases from a PTC tend to be multiple and mostly afflict the ribs, vertebrae, and sternum [11]. The incidence of FTC is less common when compared to a PTC, but it is still more likely to spread to the bones [12]. Our patient presented with a follicular variant of a papillary thyroid cancer (FV-PTC) which amalgamates the histological and clinical presentation of both subvariants of a DTC, however, FV-PTC has more resemblance with a follicular carcinoma [10].

As already stated, a thyroid malignancy usually presents as a lone neck mass. It is rare for thyroid cancers to first come to attention with symptoms of a metastatic disease [13]. Our patient demonstrated a well-differentiated and encapsulated FV-PTC with distant metastasis as his initial presentation, and we wish to highlight the rarity of such a clinical exhibition. Calvarial metastases are uncommon, and rarely breach the inner table to involve the dura mater. Making a diagnosis can be especially challenging because of the exceptionally rare occurrence of this clinical entity which may lead some clinicians to erroneously diagnose it as another lesion, such as a meningioma. Metastatic lesions to the skull are mostly asymptomatic masses that usually colonize to the occipital region. However, our case showed the involvement of the vertex which is another unusual clinical find. Per our review of the literature, a pathological fracture of the humerus, as in our case-report, is also very uncommon with only one other article reporting a similar finding due to a DTC with follicular metastatic cancer [14].

Diagnosing such a clinical entity is based on maintaining clinical suspicion and obtaining consequent radiological evidence. Imaging with a CT scan or MRI is essential for detecting the lesion, localizing the primary pathology as well as assessing the extension of the disease process. Lesions infiltrating into the bones may appear osteolytic on an X-ray and/or a CT scan. They usually have a very prominent vascular component which is analogous to the findings in our patient. These tumors may have a dense blood supply and an angiography may be necessary to assess the vascularity and guide resection [3]. We followed the conventional approach for the management of a metastatic PTC which includes a total thyroidectomy, along with the removal of any resectable metastatic lesions followed by radioactive iodine (RAI) and/or external beam radiation at the sites of metastases. For cases where the metastatic disease is found to be resistant to conventional therapies, some clinical trials show promise with the use of anti-angiogenic tyrosine kinase inhibitors (TKIs) such as Sorafenib [15-17]. Dismayingly, this all-encompassing treatment plan yields unsatisfactory outcomes because the prognosis for such patients continues to be dissatisfactory, with a 10-year survival rate of only 27% [18].

We conducted a literature review of all the cases between 1986 and 2018 using PubMed. To date, 21 cases of PTC with skull metastasis have been reported (Table 1). The mean age of these patients was 60.1 years, ranging from 25 to 76 years. A female predominance (71%) was observed in patients presenting with this disease process. Per our review, eight of the 21 cases had the follicular variant of PTC which was similar to the diagnosis in our case. We noted that dural involvement with skull metastases was only found in nine previous cases, with our case as the tenth. This reiterates the rarity of such a demonstration. Half of the patients underwent a surgical resection for their bone metastases while others were subjected to different treatment modalities which included RAI, external beam irradiation via intensity-modulated radiotherapy (IMRT) and radiotherapy. Patient demise was reported in two of these cases while the remaining patients have had a favorable clinical outcome to date.

**Table 1:** Literature review of all the cases between 1986 and 2018 of PTC with skull metastasis, utilizing PubMed. M: Male; F: Female; PTC: Papillary thyroid carcinoma; RAI: Radioactive iodine; IMRT: External beam irradiation via intensity modulated radiotherapy.

PUBLICATION	AGE	SEX	CLINICAL PRESENTATION	MICROSCOPIC FEATURES	SITE OF METS	DURAL INVOLVEMENT	TREATMENT	OUTCOME
Nigam [3]	48	F	Painless swelling on the occipitoparietal region of the scalp	PTC	Occipitoparietal region with intracranial extension	Yes	Chemotherapy, Radiotherapy	Uneventful
Li [9]	61	F	Asymptomatic	PTC	Frontal and Parietal skull	Yes	Surgery, RAI	Uneventful
Tunio [19]	74	F	Left upper neck pain, CN XII palsy.	PTC, Tall cell variant	Left occipital condyle and left side of clivus	No	IMRT	Uneventful
Tunio [19]	67	F	Headache, diplopia, facial weakness, CN II, III and VI palsy.	PTC, Insular variant	Right cavernous sinus extending to the pituitary fossa and clivus	Inconclusive	IMRT	Uneventful
Tunio [19]	65	M	Headache, dysphagia, hoarseness, dysarthria, hearing impairment, CN IX, X, XI, XII palsy.	PTC, Follicular variant	Left temporal bone with intracranial extension	Inconclusive	Surgery, IMRT	Uneventful
Jouhar [20]	42	F	Lower back pain with fever and sweating	PTC, Follicular variant	Parietal skull, Sacrum	No	Palliative management	Unknown
Al-Qahtani [21]	65	F	Painful lump over the occipital region	PTC, Follicular variant	Left occipital bone with intracranial invasion	Yes	IMRT, Sorafenib	Uneventful
Sisson [22]	65	M	Posterior head swelling	PTC, Follicular variant	Posterior skull	Yes	RAI, Thyroxine therapy	Uneventful
Pyo [23]	25	M	Asymptomatic	PTC, Follicular variant	Frontal bone and the left 5th rib	No	Surgery, RAI, Palliative radiation	Uneventful
Freeman [24]	50	M	Facial pain, proptosis, Horner syndrome.	PTC	Sphenoid/Ethmoid sinus, skull base	Yes	IMRT, RAI	Uneventful
Kung [25]	55	F	Mass in right occipital region	PTC	Occipital bone	No	Surgery, Radiotherapy	Uneventful
Feng [26]	62	F	Headache, painless mass in forehead	PTC	Frontal bone	Yes	Surgery	Uneventful
Kusunoki [27]	70	F	Left Parietal mass	PTC	Parietal bone	No	Surgery	unknown
Hugh [28]	64	F	Mass in lateral skull base	PTC, Follicular variant	Occipital bone, Petrous bone, cerebellopontine angle	Inconclusive	Surgery, IMRT	Uneventful
Kutluhan [29]	61	M	Left postauricular swelling, Multiple CN palsies	PTC	Temporoccipital region	No	RAI, Radiotherapy	Unknown
Houra [30]	76	F	Headache, confusion, Painful mass on right side of forehead	PTC	Frontal bone extending intracranially	Yes	Surgery	Uneventful
Miyawaki [31]	55	F	Asymptomatic neck mass	PTC, Follicular variant	Parietal bone, Lung	No	Surgery, Radiotherapy	Uneventful
Mostarchid [32]	50	F	Headache, torticollis, scalp mass	PTC	Temporoparietal occipital region	Yes	Refused treatment	Died
Hashiba [33]	74	F	Painless mass on the forehead, café au lait spots on the body	PTC	Frontal bone	Yes	Surgery, RAI	Uneventful
Yan [34]	73	M	Headache, diplopia, visual impairment	PTC	Skull base, Clivus	No	Surgery	Died
Cardenas [35]	59	F	Asymptomatic	PTC, Follicular variant	Occipital bone, Ribs, sacrum, ischium, femoral neck	No	RAI	Uneventful
Our case	54	M	Painless frontal bone mass, right arm pain.	PTC, Follicular variant	Frontal bone and diaphysis of Humerus.	Yes	Surgery, Radiotherapy	Uneventful

## Conclusions

PTC with distant metastases markedly worsens the patient’s prognosis. It is rarely associated with a metastatic spread to the skull. Diagnosing such a rare clinical entity is a challenging task and is made possible via a high clinical suspicion and resultant radiographic evidence. Metastatic growth usually presents as an asymptomatic skull mass which is mostly located in the occipital region, but a few cases of frontal involvement have been reported. The conventional therapy for a metastatic PTC includes total thyroidectomy, the removal of resectable metastatic lesions, RAI and/or external beam radiation at the sites of metastases. Tyrosine kinase inhibitors have proven beneficial for refractory cases. This case and our literature review illustrate that skull metastases should be considered in the clinical course of PTC because early diagnosis leads to prompt treatment which can improve patient survival.
